# *Salmonella bongori* Provides Insights into the Evolution of the Salmonellae

**DOI:** 10.1371/journal.ppat.1002191

**Published:** 2011-08-18

**Authors:** Maria Fookes, Gunnar N. Schroeder, Gemma C. Langridge, Carlos J. Blondel, Caterina Mammina, Thomas R. Connor, Helena Seth-Smith, Georgios S. Vernikos, Keith S. Robinson, Mandy Sanders, Nicola K. Petty, Robert A. Kingsley, Andreas J. Bäumler, Sean-Paul Nuccio, Inés Contreras, Carlos A. Santiviago, Duncan Maskell, Paul Barrow, Tom Humphrey, Antonino Nastasi, Mark Roberts, Gad Frankel, Julian Parkhill, Gordon Dougan, Nicholas R. Thomson

**Affiliations:** 1 Wellcome Trust Sanger Institute, Wellcome Trust Genome Campus, Hinxton, Cambridge, United Kingdom; 2 Centre for Molecular Microbiology and Infection, Division of Cell and Molecular Biology, Imperial College London, London, United Kingdom; 3 Departamento de Bioquímica y Biología Molecular, Facultad de Ciencias Químicas y Farmacéuticas, Universidad de Chile, Santiago, Chile; 4 Dept. Sciences for Health Promotion “G. D'Alessandro”, University of Palermo, Palermo, Italy; 5 Department of Medical Microbiology and Immunology, School of Medicine, University of California at Davis, Davis, California, United State of America; 6 Department of Veterinary Medicine, University of Cambridge, Cambridge, United Kingdom; 7 School of Veterinary Medicine and Science, University of Nottingham, Sutton Bonington, Leicestershire, United Kingdom; 8 National Centre for Zoonosis Research, University of Liverpool, Leahurst Campus, Neston, Wirral, United Kingdom; 9 Dipartimento di Sanità Publica, Università di Firenze, Italy; 10 Institute of Comparative Medicine, Faculty of Veterinary Medicine, University of Glasgow, Glasgow, United Kingdom; Yale University, United States of America

## Abstract

The genus *Salmonella* contains two species, *S. bongori* and *S. enterica.* Compared to the well-studied *S. enterica* there is a marked lack of information regarding the genetic makeup and diversity of *S. bongori*. *S. bongori* has been found predominantly associated with cold-blooded animals, but it can infect humans. To define the phylogeny of this species, and compare it to *S. enterica*, we have sequenced 28 isolates representing most of the known diversity of *S. bongori.* This cross-species analysis allowed us to confidently differentiate ancestral functions from those acquired following speciation, which include both metabolic and virulence-associated capacities. We show that, although *S. bongori* inherited a basic set of *Salmonella* common virulence functions, it has subsequently elaborated on this in a different direction to *S. enterica*. It is an established feature of *S*. *enterica* evolution that the acquisition of the type III secretion systems (T3SS-1 and T3SS-2) has been followed by the sequential acquisition of genes encoding secreted targets, termed effectors proteins. We show that this is also true of *S. bongori*, which has acquired an array of novel effector proteins (*sboA-L*). All but two of these effectors have no significant *S. enterica* homologues and instead are highly similar to those found in enteropathogenic *Escherichia coli* (EPEC). Remarkably, SboH is found to be a chimeric effector protein, encoded by a fusion of the T3SS-1 effector gene *sopA* and a gene highly similar to the EPEC effector *nleH* from enteropathogenic *E. coli*. We demonstrate that representatives of these new effectors are translocated and that SboH, similarly to NleH, blocks intrinsic apoptotic pathways while being targeted to the mitochondria by the SopA part of the fusion. This work suggests that *S. bongori* has inherited the ancestral *Salmonella* virulence gene set, but has adapted by incorporating virulence determinants that resemble those employed by EPEC.

## Author Summary

The bacterial genus *Salmonella* consists of two species: *Salmonella enterica* and *Salmonella bongori*. *Salmonella* are common causes of food poisoning in humans and can also cause more severe disease such as typhoid fever. Most of the *Salmonella* that cause disease in humans and animals are members of *S. enterica*. On the other hand *S. bongori,* is largely associated with reptiles but can cause disease in humans, albeit rarely. We have determined genomes for *S. bongori* isolates representing its known diversity. Using this, and existing genome information for a large number of different members of *S. enterica*, we were able to identify functions found in both species, and therefore likely to be ancestral, and differentiate them from those that have been more recently acquired. This information gives us more perspective on how pathogens evolve over the longer-term and allows us to identify functions that are associated exclusively with isolates that commonly cause disease in humans. Our analysis suggests that when *S. bongori* and *S. enterica* diverged they evolved to occupy very different niches.

## Introduction

*Salmonella* serovars are predominately pathogenic *Enterobacteriaceae* that are thought to have diverged from a common ancestor with *Escherichia coli* ∼100 million years ago [1]. The genus *Salmonella* currently comprises two species; *S. bongori* and *S. enterica*, with *S. enterica* being comprised of 6 subspecies *enterica*, *salamae*, *arizonae*, *diarizonae*, *houtenae* and *indica* [2], [3], [4], [5], [6]. These *S. enterica* subspecies are further subdivided into >2500 serovars. Although *S. bongori* have been reported to infect humans [7], [8], the species is predominantly associated with cold-blooded animals whereas serovars causing disease in humans and other warm-blooded animals mostly belong to *S. enterica* subspecies *enterica.* Since *S. enterica* incorporates clinically important pathogens, our knowledge about the genus *Salmonella* is heavily biased and there is a marked paucity of information relating to the genetic and phylogenetic makeup of *S. bongori*.

Even though *E. coli* and *Salmonella* are estimated to have diverged millions of years ago, their genomes still display significant similarity including extensive regions of synteny. However, in common with other *Enterobacteriaceae* significant diversity has been driven by horizontal gene transfer on a background of gradual genome sequence drift [9]. Many of the genes which are unique to *Salmonella* serovars, compared to *E. coli,* are found on large discrete genomic islands that include prophage elements and specialised loci termed *Salmonella* pathogenicity islands (SPIs) [10], [11], [12]. These *Salmonella*-specific functions include many genes required for the full expression of virulence and some of these were acquired by *S. enterica* following the split from *S. bongori*. For example, *S. enterica* encodes two complete type III secretion systems encoded by SPI-1 (T3SS-1) and SPI-2 (T3SS-2) [13], [14], [15], [16], whereas *S. bongori* lacks SPI-2, which is required for optimal replication within macrophages [15], [17], [18].

Several characteristics of *S. bongori* suggest that this species may, in evolutionary terms, lie somewhere between *E. coli* and *S. enterica*. Consequently, to prove this hypothesis we have studied multiple genotypic and phenotypic characteristics of *S. bongori* and compared these to *S. enterica* and other *Enterobacteriaceae*. In terms of genotype, we have determined a reference quality sequence of *S. bongori* 12419, originally isolated from an African frog in 1972 (*Salmonella* Reference Collection C strain SARC11) and prepared draft sequences of a globally and temporally diverse *S. bongori* collection including 21 representatives of the 23 known serovars (SV). From our data we have been able to determine inter and intra-species phylogeny and have used this to differentiate ancestral and more recently acquired virulence and metabolic functions. These data show that *S. bongori* possesses only a basic set of ancestral *Salmonella* virulence functions and lacks several metabolic pathways that define *S. enterica.* Nevertheless, *S. bongori* has not remained functionally static; it has acquired a repertoire of 12 T3SS candidate effector proteins, 10 of which are not found in other salmonellae but are significantly similar to known effectors found in enteropathogenic *Escherichia coli* (EPEC) strains. We herein demonstrate that representatives of these effectors are translocated and that at least one of these effectors, *S. bongori* outer protein H (SboH), is functionally related to the non-LEE encoded EPEC effector NleH1.

## Results

### Phylogenetic analysis of the species *S. bongori*

**Figure 1:**
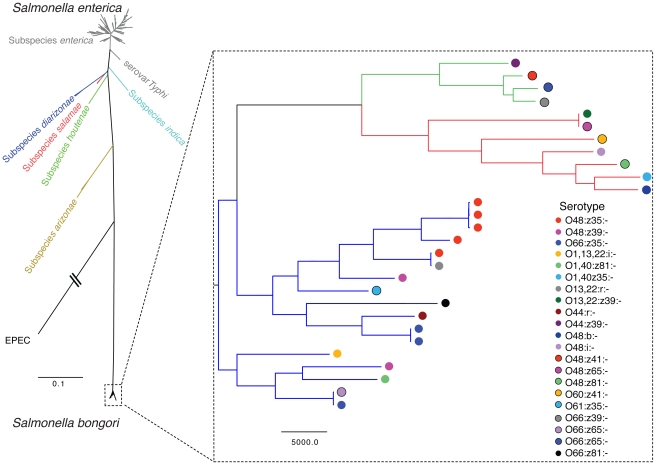
Maximum Likelihood Phylogenetic tree of *Salmonella* based on concatenated MLST loci. The relationships of the isolates shown in the enlarged region were produced using a Maximum Likelihood phylogenetic tree of *S. bongori* based on the whole genome alignments produced by mapping sequence reads to the reference genome *S. bongori* 12419 (see [82]). The location for the root for the tree for the enlarged region was determined by using *S. arizonae* as an outgroup. The *S. bongori* isolates shown represent 21 different serotypes (SV), inferred by the coloured circles, the tree branches are coloured by BAPS cluster (SNP counts for each branch and strain labels for each node are in Figure S1).

To place *S. bongori* in the context of *S. enterica* we produced a phylogenetic tree using the concatenated MLST gene sequences (as described in [19]) from a selection of *S. enterica* Sequence types (STs) covering all of the subspecies of *S. enterica*. The STs for *S. enterica* were obtained from the *S. enterica* MLST website (mlst.ucc.ie). The *S. bongori* MLST gene sequences were extracted from our sequenced strains (described in Table S1), and the EPEC MLST gene sequences were extracted from the genome sequence of strain E2348/69 (Figure 1). Despite the spatial, temporal and phenotypic diversity described within our collection, the *S. bongori* species forms a surprisingly tight cluster of sequence types (STs) clearly separated from the *S. enterica* subspecies (Figure 1). The *S. bongori* isolates in our collection fall into 20 STs, which include 11 novel *Salmonella* STs (currently *S. bongori-*specific; Table S1). In comparison, there were 1,419 STs identified as being part of *S*. *enterica* present in the MLST database as of the 3^rd^ of May 2011. To investigate the diversity and population structure of *S. bongori* we finished and fully annotated the genome of *S. bongori* 12419 (also known as SARC11 [20]). We then used this genome as a reference to produce whole genome sequences for our collection of 27 further *S. bongori* isolates. Using the whole genome sequences we produced a phylogenetic tree using RAxML (Figure 1), following the removal of mobile genetic elements (MGE; regions excluded from this analysis are listed in Table S2). In order to determine the branch on which the root should be placed we also completed a separate mapping including S. *enterica* subspecies *arizonae* strain CDC346-86 (*S. arizonae*; EMBL CP000880) strain in order to provide an outgroup. When using *S. arizonae* to locate the root for the *S. bongori* tree at least three phylogenetic clusters are evident, a feature that is supported by a clustering analysis performed using the program Bayesian Analysis of Population Structure [21], [22]. One of these clusters appears to be basal to the other clusters, based on the position of the root. The clusters are separated by 15,948-22,398 SNPS (Figure S1). The level of SNP variation between the clusters is consistent with the level of SNP variation between two serovars of *S. enterica*. For example, 39,156 SNPs differentiate *S. enterica* subspecies *enterica* serovar Typhimurium (*S*. Typhimurium) strain SL1344 and *S. enterica* subspecies *enterica* serovar Enteritidis (*S*. Enteritidis) strain P125109 (data not shown). Within *S. bongori* serotype does not appear to provide a meaningful indication of phylogenetic relationships within the population (Figure 1). This feature of the dataset may imply that there is frequent lateral gene transfer amongst *S. bongori* strains.

It is also apparent from the genomic data that there is a larger difference in the shift in genome G+C content in *S. enterica* following the divergence of the salmonellae, compared with *S. bongori* (Figure S2). Considering *S. enterica* and *S. bongori* have been evolving over the same time period these differences are remarkable. Changes in G+C content over time are thought to reflect subtle differences in mutational bias as a consequence of different lifestyles [23]. The combined data in Figure 1 and Figure S2 suggest that there has been a greater increase in G+C content accompanying the specialisation of *S. enterica* subspecies into warm-blooded hosts.

### Genetic flux across the salmonellae

**Figure 2:**
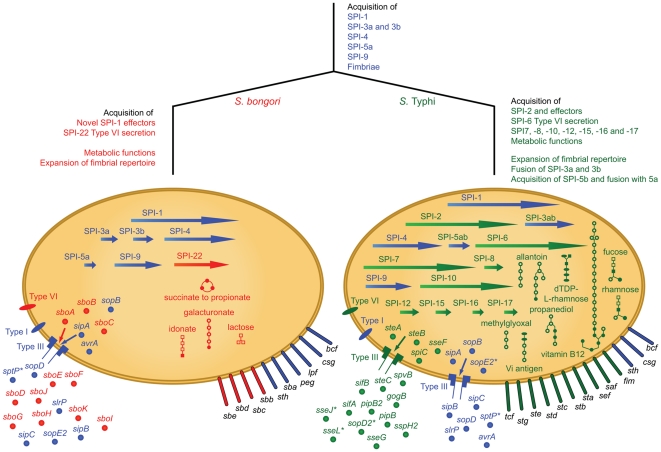
Events in the evolutionary history of *Salmonella bongori* and *S.* Typhi, a phenotypically and evolutionarily distant member of *S. enterica*. Traits shared by the common ancestor are depicted in blue; those unique to *S. bongori* are shown in red and those unique to *S.* Typhi in green. Arrows, *Salmonella* Pathogenicity Islands (SPIs); extended ovals, fimbriae; circles, effectors; small ovals and needle complexes, secretion systems. Metabolic pathways: lines, enzymatic reactions; open squares, carbohydrates; ovals, pyrimidines; open circles, other substrates; filled shapes, phosphorylated. Novel effectors acquired by *S. bongori* are secreted by the type III secretion system encoded on SPI-1. SPI-3a and 3b carry the same genes in both organisms but are fused into one island in *S.* Typhi. SPI-5a also carries the same genes in both organisms, but a further 3 kb (termed SPI-5b) has fused to SPI-5a in *S.* Typhi. *indicates a pseudogene.

To obtain a comprehensive view of genetic flux over time we used data from the other available *Salmonella enterica* genome sequences, along with our 28 sequenced *S. bongori* isolates. To complement this analysis, we used a pan-*Salmonella* microarray [24], which included *S. bongori-*specific probes, to look at gene presence/absence across the SARC collection where whole genome sequences are lacking (see methods). First we focussed our analysis on virulence functions that unified or distinguished *S. bongori* from the other salmonellae (summarised in Figure 2). Functions discussed below are conserved amongst all 28 *S. bongori* strains we sequenced (unless otherwise stated) and are not isolate-specific.

### *Salmonella* pathogenicity islands

Of the 22 reported SPIs only SPI-1, SPI-4 and SPI-9 are present in *S. bongori* 12419 with the same gene composition as those defined in *S. enterica* (Summarised in Figure 2; Table 1). Consistent with previous observations SPI-3 and SPI-5 are incomplete: SPI-3 exists as two independent insertions in *S. bongori,* SPI-3a and SPI-3b, that appear to have fused into a single element in *S. enterica* (Figure 2; This study; [25]). SPI-5 has previously been shown to be a chimeric genomic island composed of two regions of markedly differing G+C content in *S. enterica*, region one carrying the T3SS-1 translocated effector genes *sigE, sopB* and *pipD* and region 2 encoding the T3SS-2 translocated effector gene *pipB*. *S. bongori* possesses region 1 only; there is no trace of the T3SS-2 effector gene encoded in region 2.

**Table 1:** Distribution of known SPIs in the salmonellae.

	*In silico* genome analysis						Microarray data of *S. enterica* subspecies						
							SARC electrophoretic groups&						
Genomic Island	*S. bongori 12419*	S. Typhi CT18	*S*. Typhimurium LT2	S. Enteritidis P125109	S. Gallinarum 287/91	S. Cholerasuis SC-B67	II	IIIa	IIIb	IV	V	VI	VII
SPI-1	+	+	+	+	+	+	+	+	+	+	+	+	+
SPI-2	−	+	+	+	+	+	+	−	+	−	V	+	−
SPI-3	%	+	+	+	+	+	+	V	V	V	+	+	V
SPI-4	+	+	+	+	+	+	+	−	−	+	+	+	+
SPI-5	£	+	+	+	+	+	NFR	NFR	NFR	NFR	NFR	NFR	NFR
SPI-6	−	+	+	+Ψ	+Ψ	+	V*	V*	V*	V*	−	V*	V*
SPI-7	−	+	−	−	−	−	−	−	−	−	−	−	−
SPI-8	−	+	−	−	−	−	−	−	−	−	−	−	−
SPI-9	+	+	+	+	+	+Ψ	+	+	+	+	+	+	V
SPI-10	−	+	−	+Ψ	+Ψ	−	−	−	−	−	−	−	−
SPI-11	V	V	V	V	V	+	NFR	NFR	NFR	NFR	NFR	NFR	NFR
SPI-12	−	+	+	+	+	+	V	V	V	V	V	V	V
SPI-13	−	−	+	+	+	+	−	+	+	V	−	−	+
SPI-14	−	−	+	+	+	+	−	+	−	+	−	−	+
SPI-15	−	+	−	−	−	−	−	−	−	−	−	−	−
SPI-16	−	+	+	+	+	V	−	−	−	−	−	−	−
SPI-17	−	+	−	+	+	−	−	−	−	−	−	−	−
SPI-18	−	+	−	−	−	−	−	−	+	−	−	−	−
SPI-19	−	−	−	+Ψ	+	−	+	−	−	−	−	−	−
SPI-20	−	−	−	−	−	−	NT	+ $	NT	NT	NT	NT	NT
SPI-21	−	−	−	−	−	−	NT	+ $	NT	NT	NT	NT	NT
SPI-22	+	−	−	−	−	−	−	−	−	−	−	−	−

+, all genes are conserved; -, entire SPI is missing; V, partially present; Ψ, pseudogenes are contained on SPI;

*saf BCD only; %, SPI-3 present as two independent genomic islands in *S. bongori*: SPI-3A and SPI-3B (see text for details); £, only half of this island is represented in *S. bongori* (see text for details); NFR, detected but not fully represented on array; NT, not tested; $, taken from [37];

& taken from [20]; SARC electrophoretic groups (EG) relate to the following subspecies: *S. enterica* subsp. *salamae* - EG II; *S. enterica* subsp. *arizonae* - EG IIIa; *S*. *enterica* subsp. *diarizonae* - EG IIIb; *S. enterica* subsp. *houtenae* - EG IV & VII; *S. bongori* - EG V; *S. enterica* subsp. *indica* - EG VI. Microarray data was submitted to ArrayExpress under accession number E-TABM-931.

A significant distinguishing feature of *S. bongori* is the lack of SPI-2 [14], [18], [26], [27]. The site occupied by SPI-2 in *S. enterica* (alongside tRNA*-valV*) carries a ∼20 kb genomic island in *S. bongori* encoding a novel type VI secretion system (SPI-22; see below). The tetrathionate respiration (*ttr*) gene cluster which lies alongside SPI-2 in *S. enterica* is retained by *S. bongori*.

All the T3SS-2 translocated effectors are absent from *S. bongori* with the exception of SlrP, which in *S. enterica* subspecies is known to be secreted by both T3SS-1 and T3SS-2 [28]. Conversely 10 of the 12 known T3SS-1 translocated effectors are almost entirely conserved between *S. enterica* subspecies and *S. bongori* and include those that stimulate proinflammatory responses, bind actin and are important for cellular invasion (*sipA, sipB, sipC, sopB, sopD and sopE2*). *S. bongori* also carries effectors that dampen down cytoskeletal rearrangements and host signalling responses by *S. enterica* subspecies including *avrA* (reported to inhibit NF-kappa B [29]) and *sptP* (pseudogene). Although the *S. bongori sopA* gene is located at the same site as its orthologues within *S. enterica* it has been disrupted by an insertion which has generated a chimeric effector protein (denoted SboH; see below).

The *S. bongori* T3SS-1 translocated effector genes are found at exactly the same genomic loci as they are in *S. enterica*: carried on SPI-1 itself, SPI-5 or at identical sites in the chromosomal backbone (Table 2). This suggests that most T3SS-1 effectors were sequentially acquired prior to speciation, *sopE* and *sspH1* being the only exceptions. The latter two effector proteins are sporadically distributed in *S. enterica* subspecies *enterica* isolates, and carried as cargo on phage [30], [31], consistent with them being more recent acquisitions.

**Table 2:** Distribution of type three secretion systems encoded by SPI-1 (T3SS-1) and SPI-2 (T3SS-2) and their secreted effectors in the salmonellae.

			*Salmonella* species/serotypes						
			S.*bongori 12419*	*S.* Typhi CT18	*S.*Typhimurim LT2	*S.* Enteritidis P125109	*S.* Gallinarum 287/91	*S.*Cholerasuis SC-B67	*S. arizonae CDC346-86*
**Secretion system**			**T3SS effector systems**						
			**Presence/absence**						
T3SS-1			+	+	+	+	+	+	+
T3SS-2			−	+	+	+	+	+	+
			**T3SS effector proteins**						
**Genomic Location**	**Secreted by**	**Gene name**	**Presence/absence**						
øGifsy-1	SPI-2	*gogB*	−	−	+	−	−	+	−
SPI-5	SPI-2	*pipB*	−	+	+	+	+	+	−
ROD	SPI-2	*pipB2*	−	+	+	+	+ Ψ	+	−
BB	SPI-2	*sifA*	−	+	+	+	+	+	+
BB	SPI-2	*steA*	−	+	+	+	+	+	−
BB	SPI-2	*sifB*	−	+	+	+	+ Ψ	+	+
BB	SPI-2	*steB*	−	−	+	+	+	+	−
BB	SPI-2	*steC*	−	+	+	+	+	+	−
BB	SPI-1 & 2	*slrP*	+	+ Ψ	+	+ Ψ	+ Ψ	+ Ψ	+
BB	SPI-1	*sopA*	−*	+ Ψ	+	+	+ Ψ	+ Ψ	−
SPI-2	SPI-2	*spiC* (*ssaB*)	−	+	+	+	+	+	+
SPI-5	SPI-1	*sopB (sigD)*	+	+	+	+	+	+	+
BB	SPI-1	*sopD*	+	+	+	+	+	+	+
BB	SPI-2	*sopD2*	−	+ Ψ	+	+	+	+	+
øSopE and øSE12	SPI-1	*sopE*	−	+	−	+	+	−	−
BB	SPI-1	*sopE2*	+	+ Ψ	+	+	+	+	+
SPI-1	SPI-1	*avrA*	+	−	+	+	+	−	−
SPI-1	SPI-1	*sptP*	+ Ψ	+	+	+	+	+	+ Ψ
SPI-1	SPI-1	*sipA (sspA)*	+	+	+	+	+	+	+
SPI-1	SPI-1	*sipB (sspB)*	+	+	+	+	+	+	+
SPI-1	SPI-1	*sipC (sspC)*	+	+	+	+	+	+	+
SPI-2	SPI-2	*sseF*	−	+	+	+	+	+	+
SPI-2	SPI-2	*sseG*	−	+	+	+	+	+	+
BB	SPI-2	*sseL*	−	+	+	+	+	+	+
øGifsy2	SPI-2	*sseI (srfH)*	−	−	+	+	−	+ Ψ	+
BB	SPI-2	*sseJ*	−	+ Ψ	+	+	+	+	+
ROD	SPI-2	*sseK1*	−	−	+	+	+ Ψ	+	+
ROD	SPI-2	*sseK2*	−	−	+	−	−	−	−
øSE20	-	*sseK3*	−	−	−&	+	−	−	−
ROD	SPI-1 & 2	*sspHI*	−	−	+@	−	−	−	−
SPI-12	SPI-2	*sspH2*	−	+	+	+	+ Ψ	+ Ψ	+
pSLT plasmid	SPI-2	*spvB*	−	−	+	+	+	−	+

+, present; -, absent; Ψ, pseudogene;

*, see *sboH*: a chimera of sopA and a Non-LEE-encoded Type III secretion system effector gene *nleH1*; BB, chromosomal backbone; ROD, region of difference/genomic island;

@, of limited distribution in S. Typhimurium [89]; &, carried on phage ST64B of other S. Typhimurium strains [90].

In addition to the lack of SPI-2, *S. bongori* lacks the entirety of SPI-6 (encoding a type VI secretion system), SPI-13 (required for survival in chicken macrophages), SPI-14 (encoding an electron transport system) and SPI-16 (bacteriophage remnant carrying genes associated with LPS modification) making these islands unique to *S. enterica* (This study;[10], [32], [33]). From the *in silico* analysis and microarray data it is evident that SPI-6 and SPI-16 are present in all *S. enterica* lineages whilst SPI-13 and SPI-14 are only sporadically distributed in *S. enterica* (Table 1; This study [33], [34], [35]). *S. bongori* also lacks part of the centisome 54 island (CS54) encoding *shdB ratC* and *ratB* which are associated with survival in macrophages and longterm shedding of bacteria from the host [36].

### SPI-22: a novel SPI encoding a Type VI secretion system (T6SS)

**Figure 3:**
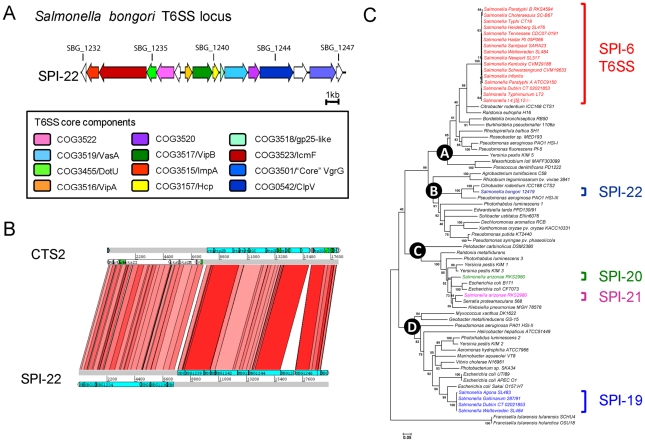
*Salmonella bongori* harbours a novel and phylogenetically distinct T6SS. **A**. Schematic representation of the SPI-22 T6SS locus. Coding sequences are represented as blocked arrows showing the direction of their transcription. Conserved core T6SS components are represented with a different color. **B.** DNA-based comparison of the T6SS encoded in SPI-22 and the CTS2 locus of *Citrobacter rodentium* strain ICC168. The analysis was performed by TBLASTX with WebACT and visualized with ACT software. **C.** Evolutionary relationships of *Salmonella* T6SS loci. A distance tree (neighbour-joining) was calculated from concatenated VipA and VipB protein sequences of previously identified T6SS gene clusters, including the novel SPI-22 T6SS locus. Each of the four major phylogenetic groups is shown in the nodes labeled A to D. Bootstrap support values (% from 3,000 replicates) were: A, 99%; B, 80%; C, 99% and D, 99%. For brevity species or serovar names are used only.

There are four distinct T6SSs currently described for *Salmonella,* encoded on SPI-6, SPI-19, SPI-20 and SPI-21 [37], [38]. *S. bongori* lacks all four systems but carries a novel T6SS locus (∼20 kb in size) which we have denoted SPI-22 (Figure 3A). The T6SS genes carried on SPI-22 shares extensive similarity to the recently identified CTS2 T6SS locus of *Citrobacter rodentium* ICC168 [39] and the HSI-III locus of *Pseudomonas aeruginosa* strain PA01 known to be required for virulence (Figures 3B & 3C) [40]. SPI-22 encodes all of the core T6SS components including homologues of DotU and IcmF, necessary for secretion and membrane stabilisation of the T6SS apparatus, the ATPase ClpV, thought to provide energy to the system, as well as other essential functions associated with the T6SS apparatus including VgrG, Hcp and the Gp25-like protein (Figure 3A)[41], [42].

### Novel T3SS effector proteins encoded by *S. bongori*

Apparently in contrast to *S. enterica,* in the absence of SPI-2 *S. bongori* has significantly expanded its repertoire of T3SS-1 effector proteins. Most of these candidate effectors are novel within *Salmonella* but are related to non-locus of enterocyte effacement (LEE) encoded (Nle) effector proteins found in EPEC, enterohemorrhagic *E. coli* (EHEC) or *C. rodentium* [39], [43]. These three enteric pathogens have a related infection strategy as they colonize the intestinal mucosa while causing attaching and effacing (A/E) lesions (reviewed by [44]). Of the 12 candidate T3SS-1 effector proteins of *S. bongori,* SboD, SboE, SboF and SboG show significant sequence similarity to NleI/G (Table 3)[45]. In addition SboC shares 57% amino acid identity with EspJ [46]. Only *sboD* and *sboC* genes have homologues in *S. enterica* subspecies: *sboD* is similar to an uncharacterized gene, *STY1076*, that is carried as ‘cargo’ on the *S.* Typhi prophage 10 [10], [47] and SboC shares 77% amino acid identity with the predicted product of *SARI_00261,* present at the same locus in *S. arizonae*.

**Table 3:** Novel candidate T3SS effector proteins.

Novel T3SS effector			*S. enterica subspecies and serovars*						
Genomic Location	Gene name	Protein family	*S. bongori* 12419	*S.* Typhi CT18	*S.* Typhimurium LT2	*S*. Enteritidis P125109	*S.* Gallinarum 287/91	*S.* Cholerasuis SC-B67	*S*. *arizonae* CDC346-86
BB	*sboH*	*sopA-nleH1* chimera	*SBG_1891*	−	−	−	−	−	−
øSB100	*sboA*	*sopA*	*SBG_0925*	−	−	−	−	−−	−
ROD	*sboB*	*sopA*	*SBG_0789*	−	−	−	−	−	−
ROD	*sboC*	*espJ*	*SBG_2380*	−	−	−	−		*SARI_00261*
ROD	*sboD*	*nleI/G*	*SBG_2043*	*STY1076*	−	−	−	−	−
Degenerate ø	*sboE*	*nleI/G*	*SBG_0673*Ψ	−	−	−	−	−	−
øSB100	*sboF*	*nleI/G*	*SBG_0924*	-	−	−	−	−	−
øSB101	*sboG*	*nleI/G*	*SBG_0932*	−	−	−	−	−	−
ROD	*sboI*	LRP	*SBG_1086*	−	−	−	−	−	−
ROD	*sboJ*	LRP	*SBG_1076*	−	−	−	−	−	−
ROD	*sboK*	LRP	*SBG_0970*	−	−	−	−	−	−
ROD	*sboL*	LRP	*SBG_0969*	−	−	−	−	−	−

LRP, leucine rich repeat protein, BB chromosomal backbone; ROD region of difference/genomic island; προπηαγε. Ψ, pseudogene.

*S. bongori* also harbours the first recognized *Salmonella* chimeric T3SS effector gene, *sboH*; a fusion of the 5′ 450 bps of *Salmonella sopA* to the 3′ 828 bps of a gene highly similar to the T3SS effector *nleH1* from EPEC, EHEC and *C. rodentium* (Figures S3 and S4). The *sboH* gene is found at the same locus as the *S. enterica sopA* gene and so is likely to have been formed by the insertion and partial deletion of *sopA* by an *nleH1* homologue. By homology the *nleH1* portion of the gene is also incomplete, lacking the first 5′ 54 bps. The most obvious effect of this fusion is to replace the cognate export signal of NleH1 (located in the N-terminal 19 amino acids [48]) with the export signal and InvB chaperone-binding site of SopA (located in the N-terminal 45 amino acids [49]) (Figure S3).

The loss of the *sopA* gene may be compensated for by the presence of two other *sopA*-related CDSs (Table 3): the product of *sboA* shares 89% amino acid identity over its full length with SopA, including the export signal, the chaperone binding domain, the invariant cysteine residue and other sites conserved in the C-terminus of this family of proteins (Figure S5 [50]). The sequence conservation between SboB and SopA is limited to the N-terminal 130 amino acids (Figure S3). The remainder of the sequence of SboB is weakly similar to a number of proteins of unknown function from a range of organisms including: *S. enterica* subsp. *arizonae* (*SARI_00821*) and *S. enterica* subsp. *enterica* serovar Kentucky (*SeKB_A1367*: Genbank ABEI01000019), other bacteria including *Providencia* and eukaryotic proteins including a protein of unknown function from *Naegleria gruberi* (Amoeba; 38.4% identity [79.1% similarity] in 211 amino acid overlap).

The remaining candidate effectors include SboI, SboJ, SboK and SboL, which all share similarity with leucine rich repeat effectors from *S. enterica,* such as SlrP, as well as Ipa invasion plasmid antigens from *Shigella*. Notably SboK is more similar to leucine rich repeat (LRP) effectors found in *Edwardsiella* and *Yersinia* spp. than SlrP.

As in many other enteric pathogens, all of the novel *S. bongori* T3SS effector genes, except *sboC*, are found on intact or degenerate prophage or regions unique to *S. bongori* compared to other salmonellae. The exception being *sboC* which is found on a backbone region conserved only in *S. arizonae* (Table 3).

### Functional analysis of the novel *S. bongori* effector proteins

**Figure 4:**
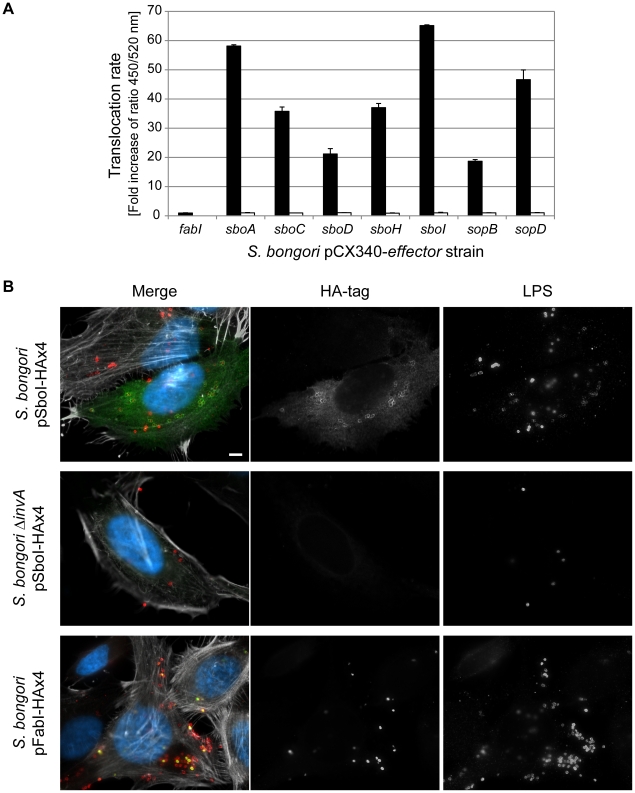
*S. bongori* translocates five novel effector proteins into the host cell. **A**. The translocation of TEM1-fusions of the putative new effectors, the positive controls SopB and SopD, and the negative control FabI into HeLa cells by *S. bongori* wild type (black bars) or the T3SS-deficient mutant *S. bongori ΔinvA* (white bars) was measured using a Fluostar Optima plate reader. The translocation rate is expressed as fold increase of the emission ratio 450/520 nm of each sample in relation to the emission ratio of uninfected cells. SboA-, SboC-, SboD-, SboH- and SboI-TEM1, along with SopB- and SopD-TEM1, but not FabI-TEM1 were translocated into the host cell. Error bars represent mean standard deviation (SD). Similar results were obtained in three independent experiments. **B**. The translocated effector SboI shows cytoplasmic distribution throughout strongly infected cells and a ring-like staining around bacteria reminiscent of a vacuolar membrane. HeLa cells were infected with *S. bongori* wild type or *ΔinvA* expressing SboI or FabI fused to four HA-tags (HAx4) for 2 h and processed for immunofluorescence microscopy (DNA - blue, HA-tag - green, LPS - red, actin - white). Bar  =  5 µm.

To confirm that the candidate T3SS effector proteins could be translocated we selected representatives of all the classes we identified (Table 3) and performed a fluorescence-based ß-lactamase translocation assay [51]. This confirmed that SboA (SopA-like), SboH (SopA - NleH1 chimera), the EspJ homologue SboC, the NleG-family effector SboD and the leucine rich repeat effector SboI were all efficiently translocated into host cells in a T3SS-1 dependent manner (Figure 4A). Translocation of the effector SboI fused to four HA-tags (HAx4) was also visualized by immunofluorescence microscopy of infected cells (Figure 4B). No translocation was observed upon infection with *S. bongori ΔinvA* expressing SboI-HAx4 or *S. bongori* wild type expressing the house-keeping protein FabI fused to the HAx4-tag. FabI-HAx4 could be detected inside a few bacteria, which was also sporadically observed for SboI-HAx4 in wild type or *ΔinvA* strains (data not shown). In contrast, upon infection with *S. bongori* wild type expressing SboI-HAx4 the effector showed cytoplasmic distribution throughout strongly infected cells, and was also found surrounding a fraction of the bacteria in a ring-like staining pattern, reminiscent of a vacuolar membrane.

**Figure 5:**
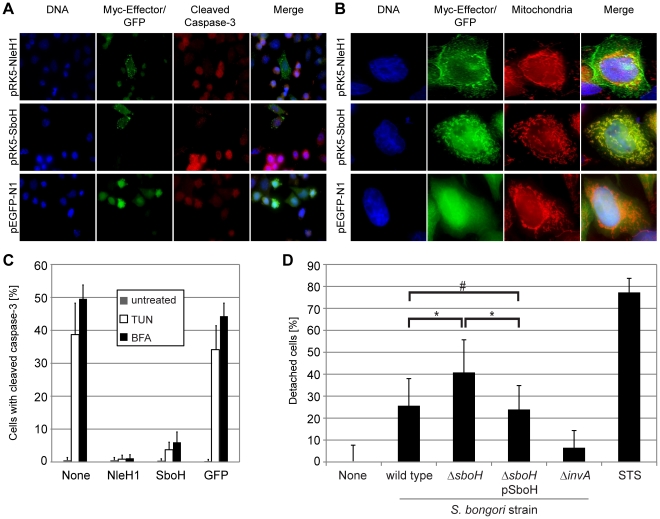
The chimeric effector SboH combines features of NleH1 and SopA and reduces host cell detachment during infection. A–C. To compare the phenotypes of SboH and NleH1 HeLa cells were transfected with pRK5-*nleH1,* pRK5-*sboH* or the control plasmid pEGFP-N1 and processed. **A**. SboH inhibits tunicamycin (TUN) and brefeldin A (BFA) induced caspase-3 activation. Immunofluorescence analysis of transfected HeLa cells treated with 5 µg/mL TUN or 10 µg/mL BFA for 18 h and stained for DNA (blue), Myc-tagged effectors (green) and activated caspase-3 (red). GFP, but not SboH and NleH1 expressing cells frequently stain positive for activated caspase-3. (Images are representative of both TUN and BFA treated cells). Bar  =  10 µm. **B.** The level of inhibition of caspase-3 activation by SboH or NleH1 was quantified by immunofluorescence counting of transfected cells. **C.** SboH and NleH1 are targeted to different subcellular locations. Transfected HeLa cells were stained for DNA (blue), Myc-tagged effector (green) and mitochondria (red) and analysed by immunofluorescence microscopy. SboH nearly exclusively co-localises with the mitochondrial marker MitoTracker, whereas NleH1 shows plasma membrane and perinuclear localisation. Scale bar  = 10 µm. **D**. Cells infected with *S. bongori* Δ*sboH* show increased cell detachment. Quantification of HeLa cells lost following 5 h infection with *S. bongori* wild type, Δ*invA* mutant, Δ*sboH* mutant and Δ*sboH* pSboH complemented strain. Staurosporine (STS) was used as a positive control to induce cell detachment. The one-way ANOVA Test using Bonferroni correction was used on data from five independent experiments and showed that the differences between *S. bongori* wild type and Δ*sboH,* as well as between *S. bongori* Δ*sboH* and the complemented strain are significant (* p-value<0.001). There was no significant difference between *S. bongori* wild type and the complemented strain (# p-value >0.05).

Since SboH is the first reported chimeric effector protein we wanted to confirm its function. The EPEC effector NleH1 was recently shown to inhibit apoptosis through a C-terminal interaction with Bax inhibitor 1 [52]. In order to determine if SboH possessed the anti-apoptotic activity of NleH1, we transfected HeLa cells with pRK5-*nleH1,* pRK5-*sboH* or a control plasmid pEGFP-N1, treated with the pro-apoptotic compounds tunicamycin (TUN) or brefeldin A and quantified the number of transfected cells showing activation of the apoptosis executioner caspase-3 by immunofluorescence microscopy. SboH prevented activation of caspase-3 by both stimuli as efficiently as NleH1 (Figure 5A & B).

The immunofluorescence analysis of transfected cells indicated that NleH1 and SboH are targeted to different subcellular locations (Figure 5A). Whereas NleH1 shows plasma membrane and perinuclear localization, SboH seemed to localize in discrete structures reminiscent of mitochondria. To substantiate this observation we stained transfected cells with the mitochondrial marker MitoTracker (Figure 5C). This demonstrated that SboH almost exclusively co-localized with the mitochondria, whereas NleH1 did not localize to the mitochondria.

To analyze the impact of SboH in *S. bongori* infection we performed a cell detachment assay as described by Hemrajani et al.[52]. This assay measures the loss of cells due to *S. bongori* infection without discriminating specific cell signaling pathways. The assay shows that a *S. bongori ΔsboH* mutant causes a moderate, but significant increase of 15% in cell loss (p-value<0.001) compared to *S. bongori* wild type (Figure 5D). Complementation of the *S. bongori ΔsboH* mutant with SboH restored levels of cell detachment to that of the wild-type.

Taken together, our data suggest that SboH combines features of SopA, namely the mitochondrial targeting signal, with the capability of NleH1 to inhibit tunicamycin and brefeldin A induced apoptosis. During infection, most likely through its anti-apoptotic activity, SboH reduces bacterial cytotoxicity and host cell loss.

### Metabolic functions mark the evolutionary divergence of *S. bongori* and *S. enterica*

We used genome sequence data to explore the distribution of metabolic pathways and their associated genes within the *Salmonella* serovars. As direct comparisons of the metabolic maps of *S.* Typhimurium and *E. coli* have been reported previously [53] we focused our analysis on comparing *S. bongori* to other *Salmonella* serovars building upon data from *S. bongori* 12419 (Figure 2). Arguably one of the most distant comparisons we could make across the salmonellae would be between *S. bongori* and the acutely pathogenic, human restricted, *S. enterica* subspecies *enterica* serovar Typhi (*S.* Typhi). Within this comparison we found a surprisingly high degree of conservation. All of the thirty pathways known to be involved in the generation of precursor metabolites and energy for *S*. Typhi are present in *S. bongori* (Table S4). Of the 146 predicted biosynthetic pathways found in *S.* Typhi, including the biosynthesis of amino acids, carbohydrates, fatty acids and lipids, only 8 are missing from *S. bongori.* Equally, of the 78 degradative pathways carried by *S.* Typhi, *S. bongori* shares 72 and possesses only 3 unique pathways.

The unique *S. bongori* metabolic capabilities (compared to *S*. Typhi) include the degradation of complex acid sugars D-galacturonate and L-idonate, which are sporadically distributed throughout the salmonellae. However, more restricted in its distribution is the capacity to degrade lactose. *S. bongori* encodes β-D-galactosidase (*lacZ*) and the lactose operon repressor (*lacI*) but is missing *lacY* (the high affinity lactose permease) which explains why although it is a non-lactose fermenter *S. bongori* gives a positive result on ortho-nitrophenyl-β-D-galactopyranoside (ONPG) medium. Most members of *S. enterica* subspecies *enterica* are phenotypically non-lactose fermenters and are unable to utilise ONPG. The only other salmonellae that appear to harbour the *lac* operon include *S. enterica* subspecies *arizonae* and *diarizonae*, but these subspecies also possess *lacY.* Comparing *S. bongori* to other *Enterobacteriaceae* which are non-lactose fermenters but are ONPG positive there is no strong conservation in genes or the site of insertion, suggesting independent acquisition events [54], [55].

There is also evidence of metabolic streamlining in *S. bongori*. For example, like *E. coli, S. bongori* has lost the *cob-pdu* gene cluster (*S. bongori* retains only fragments of the first and last gene of the *cob-pdu* cluster: *cobT* [SBG__1882] and *pduX* [SBG_1882A]) and so lacks the capacity to anaerobically synthesise vitamin B_12_ (cobalamine) and to catabolise propanediol [56]. The *cob-pdu* gene cluster was thought to have been lost by many *Enterobacteriaceae.* It has been suggested that the *cob-pdu* gene cluster was subsequently reacquired by *S. enterica,* following its split from *S. bongori* [57], [58], [59], where it has been shown to be important for survival in macrophages [60] a niche in which *S. bongori* cannot survive [61].

Conversely there is evidence that pathways conserved in *S. bongori, E. coli* and wider *Enterobacteriaceae*, have been lost and replaced in the warm blooded-host adapted serovars of *S. enterica* subspecies *enterica* by alternative pathways that are energetically more efficient producing more ATP/mol of substrate or have differing substrate specificities.

*S. bongori* carries the same genes for L-tartrate or citrate fermention as those found in *E. coli* and most other enterics: the *ttdABDT* operon or *citDEF*. However, these have been either replaced (only remnants of the *ttd* genes remain in members of subspecies *enterica* e.g. *S*. Typhi strain CT18 position 3230063..3230191 and *S*. Enteritidis strain P125109, *SEN3049A*) or, in the case of citrate, augmented in *S. enterica* subspecies *enterica* by the acquisition of two operons encoding tartrate hydratase (e.g. STM3350-3359) and a second citrate lyase gene cluster (e.g. STM0052-STM0063). Both of these clusters carry a dedicated Na^+^ translocating oxaloacetate decarboxylase to provide reducing power. Therefore unlike the pathways found in *S. bongori* and *E. coli* these new pathways do not require a co-substrate and are energetically more efficient, producing more ATP/mol of substrate [62], [63], [64]. Moreover mutations in these new gene clusters in *S.* Typhimurium can be found as attenuating in genome wide mouse mutagenesis studies [65] and our microarray analysis of SARC shows that all other *S. enterica* subspecies resemble *S. bongori* by possessing the *ttd* cluster but lacking the alternate tartrate and citrate dissimilatory operons (data not shown). Looking more broadly across the *Enterobacteriaceae,* some *Klebsiella pnenumoniae* isolates also possesses a related second citrate lyase, sometimes located at the same site in the genome as that found in *S. enterica* subspecies *enterica* serovars. However, the gene makeup of this region in *K. pnenumoniae* differs slightly and we could not find this region at this site in other enterics we searched consistent with this region being sporadically acquired.

There are other examples of lineage-specific metabolic streamlining which show a more sporadic phylogenetic distribution including the C-P lyase system (*phnA-P*), able to breakdown a wide range of phosphonate compounds, and phosphonatase which is specific for 2-aminoethylphosphonate. Whilst *S. enterica* subspecies *arizonae* carries the entire cluster (data not shown) the *S. bongori phn* loci is degenerate, consisting of only *phnOAB* and remnants of *phnP* and *phnN* (SBG_3727 and SBG_3728A, respectively).

*S. enterica* subspecies *enterica* has also lost the majority of genes in this operon leaving only *phnOAB* [66], but have acquired the phosphonatase system encoded by *phnVUTSRWX* [66]. The explanation for this replacement in members of subspecies *enterica* may lie in that fact that 2-aminoethylphosphonate is found in abundance in flagellates found in the digestive tracts of ruminants such as cattle, common hosts for members of subspecies *enterica* [67]. Our microarray data for this cluster also shows that genes *phnVUTSRWX* are only present in *S. enterica* subspecies *enterica* (data not shown).

The capacity to use allantoin as a sole nitrogen source under anaerobic conditions is also phylogenetically restricted. We have previously speculated that the acquisition of the allantoin gene cluster by *S. enterica* subsp. *enterica* was linked to differences in the sequential breakdown of purines by different hosts [68], [69]: in fish, crustaceans and other invertebrates purines are broken down sequentially to ammonia and CO_2_ [70], [71], while genetic lesions in vertebrate species block purine catabolism at different steps leading to the accumulation of allantoin in most mammals (including rodents and domesticated animals). Our current data shows that the genes encoding allantoin degradation in *Salmonella* are absent from *S. bongori* and restricted to *S. enterica* subspecies *enterica* and *salamae* only.

## Discussion

Our understanding of *Salmonella* evolution has been built largely on data from representative isolates of the relatively recently emerged *Salmonella enterica* subspecies *enterica*. *S. bongori* and *S. enterica* are thought to have diverged between 40-63.4 Myrs ago [72] and so comparing the genomes of these two distinct species provides a unique opportunity to understand the ancestral *Salmonella* and determine the evolutionary events that mark speciation and those that track different branch points in *Salmonella* evolution following this event.

We have shown that a diverse set of *S. bongori* isolates form a tight cluster of sequence types that, when examined on a whole genome basis, appear to comprise at least three phylogenetic groups. The G+C content in *S. bongori* represents a midpoint between *S. enterica* subspecies *enterica* and *E. coli* (Figure S2), but the phylogenetic analysis with MLST data and whole genome sequences suggests that this may be an artifact of the host specialisation into warm blooded animals that took place in the evolution of *S. enterica* subspecies *enterica*. The variation across the *S. bongori* species, both within and between the phylogenetic groups, contrasts sharply with that observed across *S. enterica,* with a comparable amount of variation across the groups in *S. bongori*, to that which is found between two serotypes of *S. enterica* subspecies *enterica*. The apparent lack of variation is difficult to explain since even if *S. bongori* had been stably maintained for a long period within its current niche significant genome diversity would still be expected, even if this was largely neutral. This reduced level of apparent diversity could be a bias of sampling, yet the isolates sequenced in this study are from a wide range of sources and are globally and temporally diverse. Although the answer to this question is still equivocal it is possible that *S. bongori* has been through a recent evolutionary bottleneck.

Despite their apparent evolutionary divergence, metabolic analysis showed that the biochemical maps of *S. bongori* and *S.* Typhi are very similar, suggesting that *Salmonella* serovars acquired many of the basic functions for an enteric lifestyle early in their evolution. This has been recently supported by the finding that the ability to use tetrathionate as an electron acceptor provides a competitive advantage to *S*. Typhimurium in the inflamed gut over normal flora [73]. The *ttr* cluster is conserved in *S. bongori* [74].

Where *S. bongori* did differ from *S. enterica* serovars, it generally most closely resembled *E. coli* and the wider *Enterobacteriaceae*, i.e. the presumed ancestral state. Whilst the independent acquisition of the *lac* operon by *S. bongori* is difficult to explain, for *E. coli* the acquisition of the *lac* operon may have facilitated metabolism of milk sugar and adaptation to the mammalian gut. Conversely the loss of this function from *S. enterica* subspecies *enterica* may be associated with its invasive lifestyle since recent evidence has shown that *lacI* expression interferes with the function of SPI-2 and attenuates virulence in macrophage [75].

This comparative analysis also highlighted metabolic traits that mark the evolution of *S. enterica* subspecies *enterica* including the differing abilities to ferment L-tartrate and citrate. These metabolic differences are already known to differentiate high and low pathogenicity *Salmonella* strains (*S*. Paratyphi B and *S*. Paratyphi B variant Java) [76]. Outside of *Salmonella* the ability to ferment citrate almost equally divides clinical *Klebsiella pneumoniae* biotypes into two groups and is thought to represent an adaptation to different nutrient conditions found within the host [77].

*S. bongori* possess a basic *Salmonella* virulence ‘tool kit’ consisting of SPI-1, 3a, 3b, 4, 5 and 9. Although the *S. bongori* SPI-3 and SPI-5 have a different structure compared to those in *S. enterica* these SPIs are conserved across the salmonellae and could be considered part of the *Salmonella* core genome. Moreover, many of these core SPIs show significant regulatory and functional interplay between the functions they encode. For example the SPI-4 adhesin SiiE is required for efficient translocation of T3SS-1 effectors in *S*. Typhimurium [78] and SPI-4, SPI-5 and SPI-1 genes are under joint control by the SirA/HilA global regulatory cascade [78], [79], [80], [81]. From this it is tempting to speculate that these SPIs define one of the earliest virulence networks of *Salmonella*. Clearly there are multiple factors missing from *S. bongori* which limit its ability to cause disease in warm blooded animals demonstrated by experiments that have introduced SPI-2 into *S. bongori* [61].

The genome of *S. bongori* has not remained static since divergence; we see parallels with *S. enterica* serovars in the functions that have been acquired following divergence. For example both *S. bongori* and *S. enterica* have independently acquired different T6SSs. It is clear that the *Salmonella* genus as a whole includes representatives of each of the major T6SS phylogenetic groups, thus reinforcing the long-term importance of these systems. Also like *S. enterica, S. bongori* has sequentially acquired a range of T3SS-1 effector proteins many of which we have shown to be translocated. The *S. bongori* effectors have homologues in EPEC, EHEC and *C. rodentium* including EspJ; which in EPEC and EHEC prevents receptor mediated phagocytosis of opsonised cells [46] and so could be important for *S. bongori* in resisting phagocytosis. This strategy would be well in line with previous observations of the *S. bongori* life style as the bacteria are not able to sustain an intracellular life style in macrophages mainly due to absence of the SPI-2 T3SS-2 and its effectors [61] and likely also because of the lack of *cob-pdu* operon too [60]. We also functionally characterised the effector SboH and have shown that it inhibits apoptosis in a similar manner to its EPEC homologue NleH1. Moreover, in infection SboH reduces bacterial cytotoxicity. In EPEC the anti-apoptotic activity of NleH1 has been proposed to sustain colonisation of the mucosal epithelium by reducing the ‘turn-over’ of surface enterocytes and associated any bacteria or microcolonies [52]. The acquisition of these effectors that most closely resemble those from pathogenic *E. coli* strains causing watery diarrhea suggests that following the split of *S. bongori* and *S. enterica*, *S. bongori* has adopted a specialised infection strategy which might in parts be more similar to the extracellular pathogenic *E. coli* than *S. enterica.* This infection strategy might be optimised to colonise cold-blooded reptiles, but still provides the basic armoury for *S. bongori* to emerge as an opportunistic pathogen of humans and animals.

## Materials and Methods

### Source and details of bacterial strains

The *S. bongori* exploited in this study included 28 isolates originating from between 1966-2004, from the USA, Africa and Europe, from hosts including humans, frogs, pigeons and reptiles as well as environmental sources including the shell of a hen's egg, cheese, fishmeal and waste water (see Table S1).

### Genome sequencing assembly, mapping and phylogeny

For bacterial cultures LB medium was inoculated and grown overnight at 37°C with each isolate. Genomic DNA was extracted from 1 ml of culture by using manufacturer's instructions (Wizard Genomic DNA Purification kit from Promega).

The genome of *S. bongori* strain 12419 was sequenced to approximately 11-fold coverage from pUC19 (insert size 2.8–5.5 kb) and pMAQ1b_*Sma*I (insert size 5.5–6.0 kb) genomic shotgun libraries using big-dye terminator chemistry on ABI3700 automated sequencers. End sequences from large insert BAC libraries in pBACe3.6 (insert size 23–48 kb) were used as a scaffold. All repeat regions were bridged by read-pairs or end-sequenced polymerase chain reaction (PCR) products.

For all remaining *S. bongori* strains, tagged genomic library preparation and DNA sequencing (with and without multiplexing) was carried out as previously described [82]. Mapping of reads to the reference genome and SNP detection were carried out according to earlier described protocols [82]. *De novo* assemblies were performed by using Velvet v0.7.03 and their corresponding contigs were ordered using Abacas [82]; the resulting pseudomolecules were blasted against the reference genome to assess synteny as well as the existence of indels and novel regions. Details of mapping and assembly data output are given in Table S1. Annotation and analysis was performed using Artemis and ACT [83], [84].

Phylogenic analysis of *Salmonella* (shown in Figure 1) was based on the 7 concatenated MLST loci sequences, from sequences generated in this study (for the *S. bongori* isolates), those obtained from the *Salmonella* MLST Public Strains Database (http://mlst.ucc.ie/mlst/dbs/Senterica for the *S. enterica* STs) or obtained from genomic sequence (for EPEC strain E2348/69 [43]). The *S. bongori* tree was produced using a whole genome alignment generated by mapping the *S. bongori* samples against the finished genome of strain 12419. Trees were drawn using RAxML assuming a general time reversible site model with gamma correction [85]. In the case of the whole genome tree, phage and MGEs were removed prior to the production of the tree (see Table S2). Support for nodes was assessed by using bootstrapping (x100); SNPs were reconstructed on the tree with parsimony using accelerated transformation. BAPS analysis was performed on the SNP alignments produced from the mapping alignment, using the BAPS individual mixture model [22]. Three independent iterations of BAPS were performed (using an upper limit for the number of populations of 25, 26 and 27) to obtain the most optimal partitioning of the sample.

### Defining orthologous gene-sets

To infer the orthologous genes in each pair of genomes compared: Each CDS (a) from the genome (A) was searched, using FASTA, against the CDSs of the other genome (B). If the top hit covered at least 80% of the length of both sequences with at least 30% identity, a reciprocal FASTA search of the top hit sequence (b) was launched against the CDSs of the first genome. If the reciprocal top hit was the same as the original query CDS then (a) and (b) are considered orthologous genes of (A) and (B). In a second step, in order to validate the results, we performed a BLASTN and TBLASTX comparison between the 15 genomes, visualized using ACT [83] to curate ambiguous cases, for example, gene remnants (pseudogenes), IS elements and phage-related CDSs, and to check for a syntenic relationship among the putative orthologs.

### Pathway Tools

A Pathway/Genome Database (PGDB) describing the metabolic pathways of *S.* Typhi was created in Pathway Tools v. 13.5 (SRI International, California) using the genome sequence and annotation associated with strain CT18 [10]. This PGDB underwent manual curation and currently comprises 200 predicted metabolic pathways and over 130 predicted transport reactions. To determine the differences in *S. bongori* relative to *S.* Typhi, we mapped orthologues onto the pathways and transport reactions, subsequently removing those missing functions and adding in the functions unique to *S. bongori* (summarised in Figure 2 and listed in Table S4).

### *S. bongori* deletion mutant and novel T3SS effector plasmid construction

Primers, restriction enzymes and plasmids used to create *S. bongori* deletion mutants and to construct expression vectors of the putative *S. bongori* effector proteins are listed in Table S3.

Specific gene knockouts of the *invA* or *sboH* genes were generated in *S. bongori* 12419 as described previously [86]. To create non-polar mutations, the kanamycin resistance cassette was removed using plasmid pCP20 leaving a scar of 84 bp [86], [87]. Deletions were confirmed by PCR and sequencing from the regions flanking the knockouts.

To obtain the vectors encoding ß-lactamase (TEM1) fusions all genes were PCR-amplified from *S. bongori* strain 12419 genomic DNA and PCR products digested and ligated into pCX340 [51] or pRK5, respectively. If the *Kpn*I restriction site of pCX340 was used a new ribosome binding site (RBS) was included in the forward primer. Sequence identity of the constructs was verified by DNA sequencing. The pCX340 derivative plasmids were named pICC522 (*fabI*), pICC523 (*sboA*), pICC524 (*sboC*), pICC525 (*sboD*), pICC526 (*sboI*) and pICC527 (*sboH*), pICC611 (*sopB*), pICC612 (*sopD*).

To create a plasmid allowing the C-terminal fusion of four HA-tags to the effectors, pCX340 was digested with *Eco*RI and *Xba*I to remove the *tem1* gene. Subsequently an oligo cassette encoding four HA-tags was ligated into the vector to give the plasmid pICC613. PCR products of *sboI* and *fabI* and pICC613 were digested and ligated as described for the vectors encoding TEM1 fusions to yield pICC614 (pSboI-HAx4) and pICC615 (pFabI-HAx4). The plasmid pICC616 (pSboH) allowing the inducible expression of untagged SboH was constructed by ligation of the *sboH* PCR product in *Eco*RI and *Xba*I digested pCX340 and the transfection vector pRK5-SboH (pICC548) by ligation of the PCR product into pRK5 (Clontech). All pICC plasmids were used to transform *S. bongori* strain 12419 wild type and mutant strains by electroporation.

### Translocation assay

The ß-lactamase (TEM1)-translocation assay for the identification of translocated effector proteins was adapted from a protocol previously described [51]. To obtain a confluent cell layer 4.0×10^4^ HeLa cells were seeded in 200 µL DMEM (Sigma, 1000 mg/L glucose, supplemented with 10% fetal calf serum, Glutamax (Invitrogen) and MEM non-essential amino acids (Sigma)) growth medium per well of a black wall/clear-flat bottom 96 well plate (Becton Dickinson) and cultured overnight. Prior to infection the medium was replaced with 150 µL fresh growth medium. Overnight LB broth cultures (6 µg/mL tetracycline) of *S. bongori* strain 12419 or the *ΔinvA* mutant carrying the pICC plasmids were diluted 1∶30 in LB broth (6 µg/mL tetracycline) and grown to an OD600 of 1.1–1.4 before protein expression was induced by addition of 1 mM Isopropyl β-D-1-thiogalactopyranoside (IPTG). The induced cultures were incubated until an OD600 of 1.8–2.2 was reached and diluted in Dulbecco's-PBS (D-PBS, Sigma) to a concentration of 3.75×10^8^ bacteria/mL. To infect HeLa cells 20 µL bacterial dilution per well was added and the infection was synchronized by centrifugation (900 g, 5 min). After 1 h incubation at 37°C, 5% CO_2_ the cell supernatant was replaced with 100 µL Hanks' Buffered Salt Solution (Gibco, supplemented with 20 mM HEPES, 3 mM Probenecid (Sigma) pH 7.4 designated HBSS-HP), and 20 µL freshly prepared CCF2-AM ß-lactamase substrate (LiveBLAzer FRET-B/G Loading Kit, Invitrogen) were added. After 1 h 45 min incubation at room temperature in the dark the cells were washed five times with HBSS-HP. Fluorescence emission at 450 nm and 520 nm was measured from the bottom using a Fluostar Optima plate reader (excitation wavelength 410 nm, 10-nm band-pass). The translocation rate was calculated as recommended in the LiveBLAzer FRET-B/G Loading Kit manual. Briefly, emission values were first corrected by subtraction of the average background signals recorded for empty wells and the mean 450 nm/520 nm emission ratio of a triplicate of wells was calculated for each sample. The translocation rate is expressed as fold increase of the mean emission ratio 450/520 nm of each infected sample in relation to the mean emission ratio of uninfected cells. Expression of the TEM1 fusion proteins was controlled by Western blot using a mouse anti-ß-lactamase antibody (QED Bioscience Inc; data not shown).

### Epifluorescence microscopy of translocated effectors

HeLa cells (1.25×10^5^ per 24-well plate well) were seeded on coverslips and incubated in growth media overnight in a humidified atmosphere of 5% CO_2_ at 37 °C. *S. bongori* wild type or *ΔinvA* mutant containing pICC614 or pICC614 plasmids were grown, diluted and 50 µL dilution used for infection as described for the translocation assay. 1 h45 min –2 h post infection cells were washed three times with D-PBS, fixed with 3% paraformaldehyde (PFA), treated with 50 mM NH_4_Cl in D-PBS, washed three times with D-PBS, permeabilised with 0.1% (v/v) Triton X-100, washed three times with D-PBS and blocked with 2% (w/v) bovine serum albumin (BSA) and 2% (v/v) natural donkey serum in D-PBS for 1 h. The samples were stained with rabbit anti-*Salmonella* (O:66 Statens Serum Institute) and mouse anti-HA.11 (Covance) primary antibodies followed by Rhodamine Red X (RRX)-conjugated donkey anti-rabbit IgG and DyLight 488-conjugated donkey anti-mouse IgG (both Jackson ImmunoResearch) antibodies. Nuclei were labelled with Hoechst 33342 dye and F-actin with AlexaFluor647 phalloidin (Invitrogen). The coverslips were mounted using ProLong Gold antifade reagent (Invitrogen) and analysed on Zeiss Axio Imager Z1 or M1 immunofluorescence microscopes with Axiovision Rel 4.8 software.

### Epifluorescence microscopy and caspase-3 activation assay

The experiments to compare the localization and anti-apoptotic activity of NleH1 and SboH were performed as described previously [52], [88]. Briefly, transfected HeLa cells were treated with either 5 µg/ml tunicamycin (TUN) or 10 µg/ml brefeldin A (BFA) for 18 hours, or left untreated, prior to immunofluorescence microscopy processing. The cells were fixed in 3% PFA, washed with PBS, treated with 10 mM NH_4_Cl, permeabilized with 0.2% (v/v) Triton X-100, washed with PBS and blocked with 1% (v/v) BSA in PBS for 1 h. Active caspase-3 and Myc-tagged effector proteins were detected using rabbit anti-cleaved caspase-3 (Cell Signalling Technology), RRX-conjugated donkey anti-rabbit IgG (Jackson ImmunoResearch) and Fluorescein Isothiocyanate (FITC)-conjugated monoclonal mouse anti-Myc (Sigma) antibodies. Nuclei were labelled with the Hoechst 33342 reagent (Invitrogen). Mitochondria were visualised using MitoTracker (Invitrogen) in accordance with manufacturer's guidelines before fixation. Samples were mounted and analysed by microscopy as described above. To determine the number of apoptotic cells 100 transfected cells were analysed in each repeat. Samples were tested in triplicate and experiments repeated a minimum of three times.

### Cell detachment assay

HeLa cells (7.2×10^4^ per 24-well plate well) were cultured overnight. *S. bongori* wild type, *ΔinvA*,*ΔsboH* or *ΔsboH* pICC616 were grown for infection as described above. Prior infection 1 mL bacterial culture was harvested by centrifugation, resuspended in the same volume of cell culture medium and used to infect HeLa cells. After 1 h cells were treated with 200 µg/ml gentamicin and incubated for 4 h. A control sample was incubated with 1 µM staurosporine (STS) for 5 h in parallel to the infection. Cells were washed 5 times with PBS and then trypsinized for 10 min. Trypsin was inactivated by addition of 700 µL growth medium. Cells were counted on a Neubauer hemocytometer. All counts were compared with the level of uninfected, untreated cells and plotted as a percentage of cells lost. Statistical analysis was done using the GraphPad InStat Version 3.06 software. The one-way ANOVA Test using Bonferroni correction was used to determine significance of the observed differences (p-values<0.001).

### Accession numbers

The annotated genome sequence of *Salmonella bongori* strain 12419 has been deposited in the public databases under the accession numbers FR877557. The Illumina sequencing reads for all the sequences generated in this study have been deposited in the European Nucleotide Archive (ENA) under the accession numbers ERS002029- ERS002042 (inclusive), ERS002044, ERS004246, ERS004249, ERS004170, ERS004173- ERS004176 (inclusive), ERS004190- ERS004193 (inclusive) and ERS004196. This is matched to strain names in Table S1. Microarray data was submitted to ArrayExpress under accession number E-TABM-931.

## Supporting Information

Table S1 Details of the strains sequenced in this study, the sequencing strategy, accession numbers and the estimated depth of coverage.
(XLS)

Table S2 *S. bongori* mobile genetic element locations that were excluded from phylogenetic tree.
(XLSX)

Table S3 Details of the plasmids and primers used in this study.
(XLS)

Table S4 Details of the individual pathways present or absent in *S. bongori* strain 12419 and *S*. Typhi strains CT18.
(XLS)

Figure S1 Whole genome RAxML phylogeny for *S. bongori* samples with strain labels. The tree is the same as that shown in Figure 1, but showing strain labels and the numbers of SNPs that separate each node on the tree. The two figures in brackets are nodes where bootstrap support was less than 100%.
(TIF)

Figure S2 Genomic G+C content for representatives of *E. coli*, *Shigella flexneri* and *S. enterica* and *S. bongori*.
(TIF)

Figure S3 ClustalW alignment of the protein sequences of SopA, SboH and SboB showing the regions of conservation, see key.
(TIF)

Figure S4 ClustalW alignment of the protein sequences of SboH and NleH orthologues. Systematic gene names are written down the left. EPEC strain E2348/69, enterhemorrhagic *E. coli* strain EDL933 and *Citrobacter rodentium* strain ICC168 systematic gene names begin with E2348_, ECs_ and ROD_, respectively. The regions of conservation are shown, see key.
(TIF)

Figure S5 ClustalW alignment of the protein sequences of SopA from *S.* Typhimurium strains LT2 (SopA-LT2) and *S*. Enteritidis strain P125109 (SopA-PT4) with SboA from *S. bongori* strain 12419 (SboA). Conserved ubiquitin protein ligases domain is shown, see key.
(TIF)
