# Supplementary figures and images for: Salmonella bongori Provides Insights into the Evolution of the Salmonellae
Source: PLoS Pathog. 2011 Aug 18;7(8):e1002191. doi: 10.1371/journal.ppat.1002191 (PMC3158058; doi:10.1371/journal.ppat.1002191)

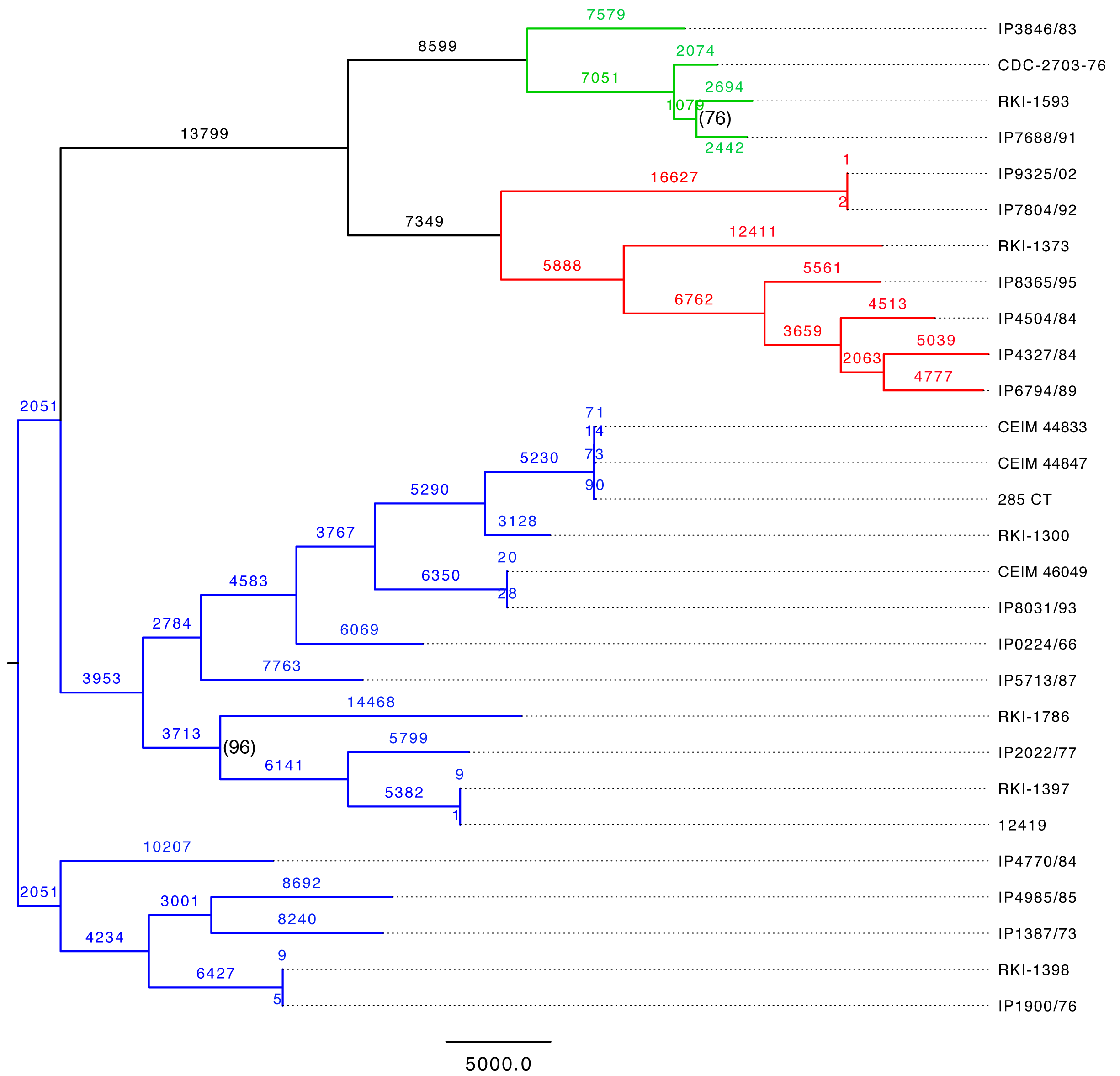

Supplement: Figure S1 — Whole genome RAxML phylogeny for S. bongori samples with strain labels. The tree is the same as that shown in Figure 1, but showing strain labels and the numbers of SNPs that separate each node on the tree. The two figures in brackets are nodes where bootstrap support was less than 100%. (TIF) [file ppat.1002191.s005.tif]

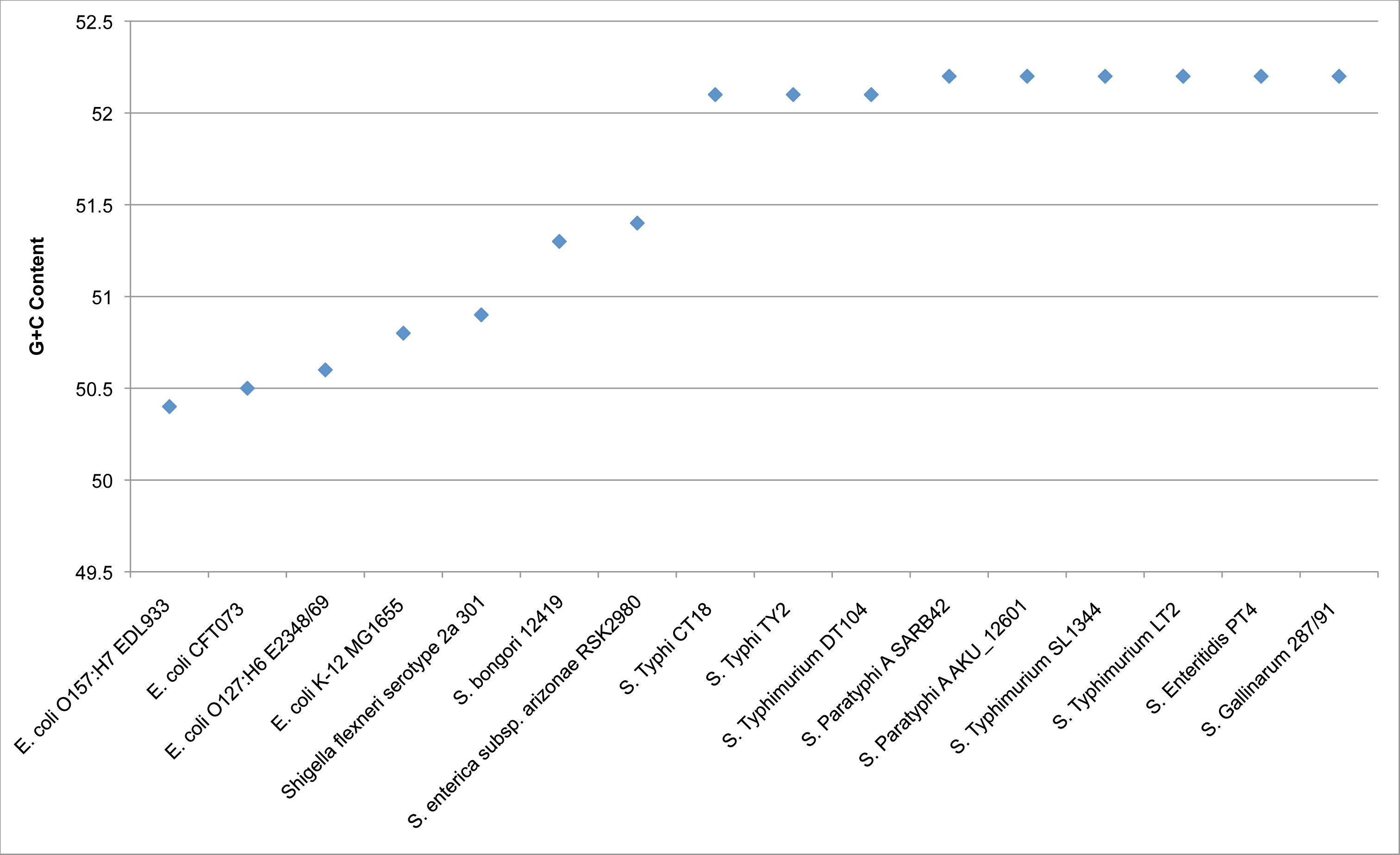

Supplement: Figure S2 — Genomic G+C content for representatives of E. coli, Shigella flexneri and S. enterica and S. bongori. (TIF) [file ppat.1002191.s006.tif]

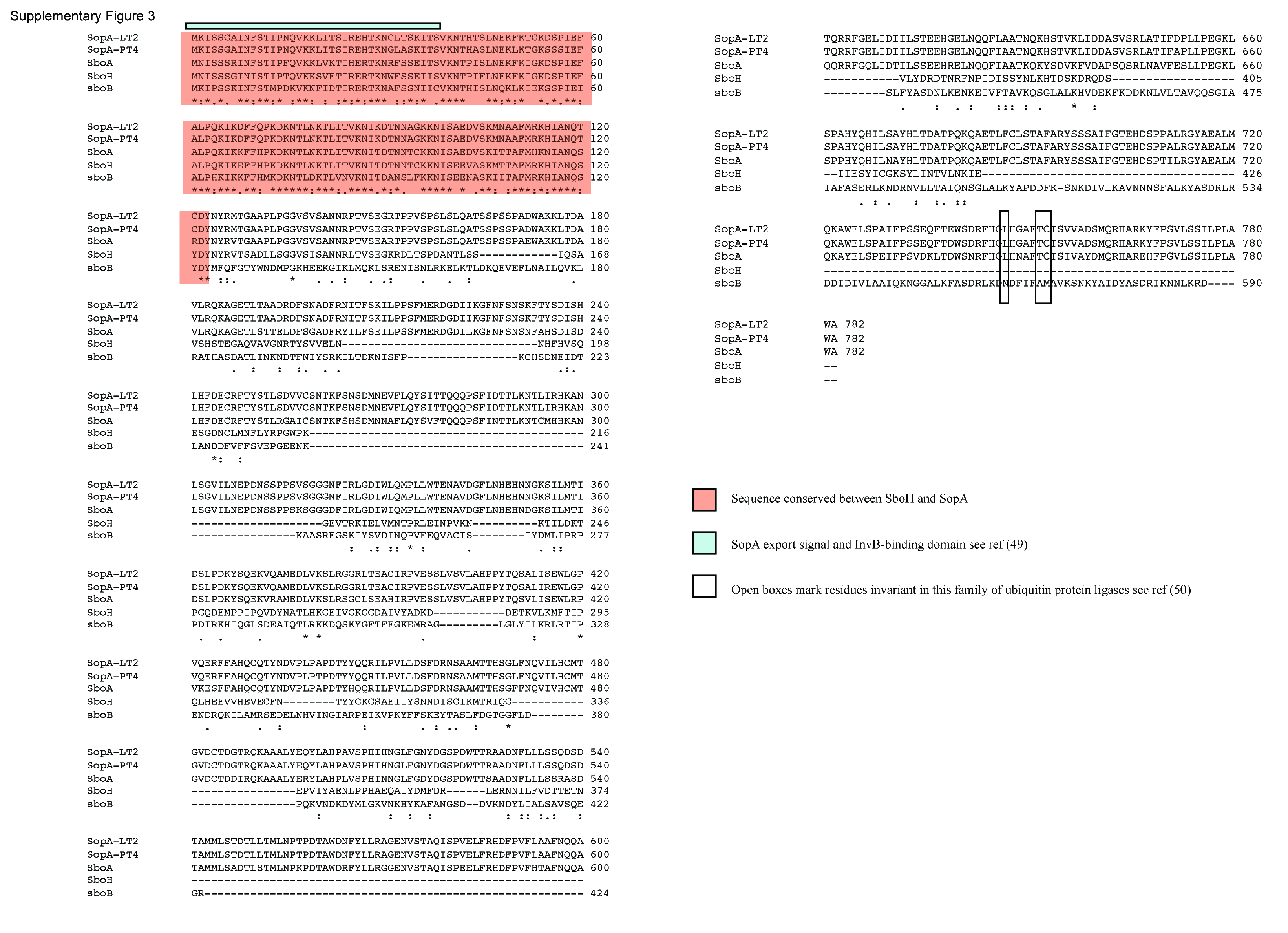

Supplement: Figure S3 — ClustalW alignment of the protein sequences of SopA, SboH and SboB showing the regions of conservation, see key. (TIF) [file ppat.1002191.s007.tif]

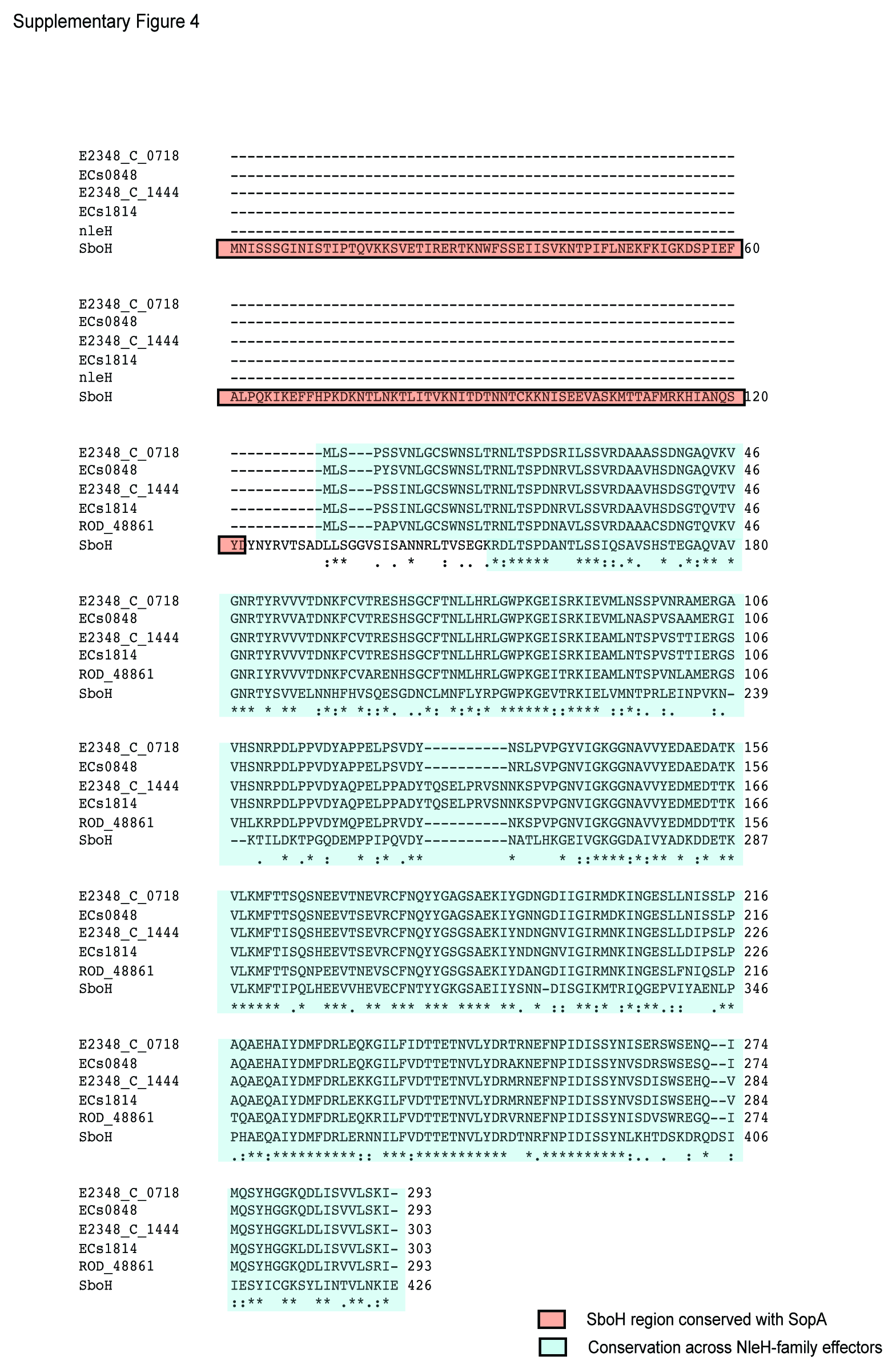

Supplement: Figure S4 — ClustalW alignment of the protein sequences of SboH and NleH orthologues. Systematic gene names are written down the left. EPEC strain E2348/69, enterhemorrhagic E. coli strain EDL933 and Citrobacter rodentium strain ICC168 systematic gene names begin with E2348_, ECs_ and ROD_, respectively. The regions of conservation are shown, see key. (TIF) [file ppat.1002191.s008.tif]

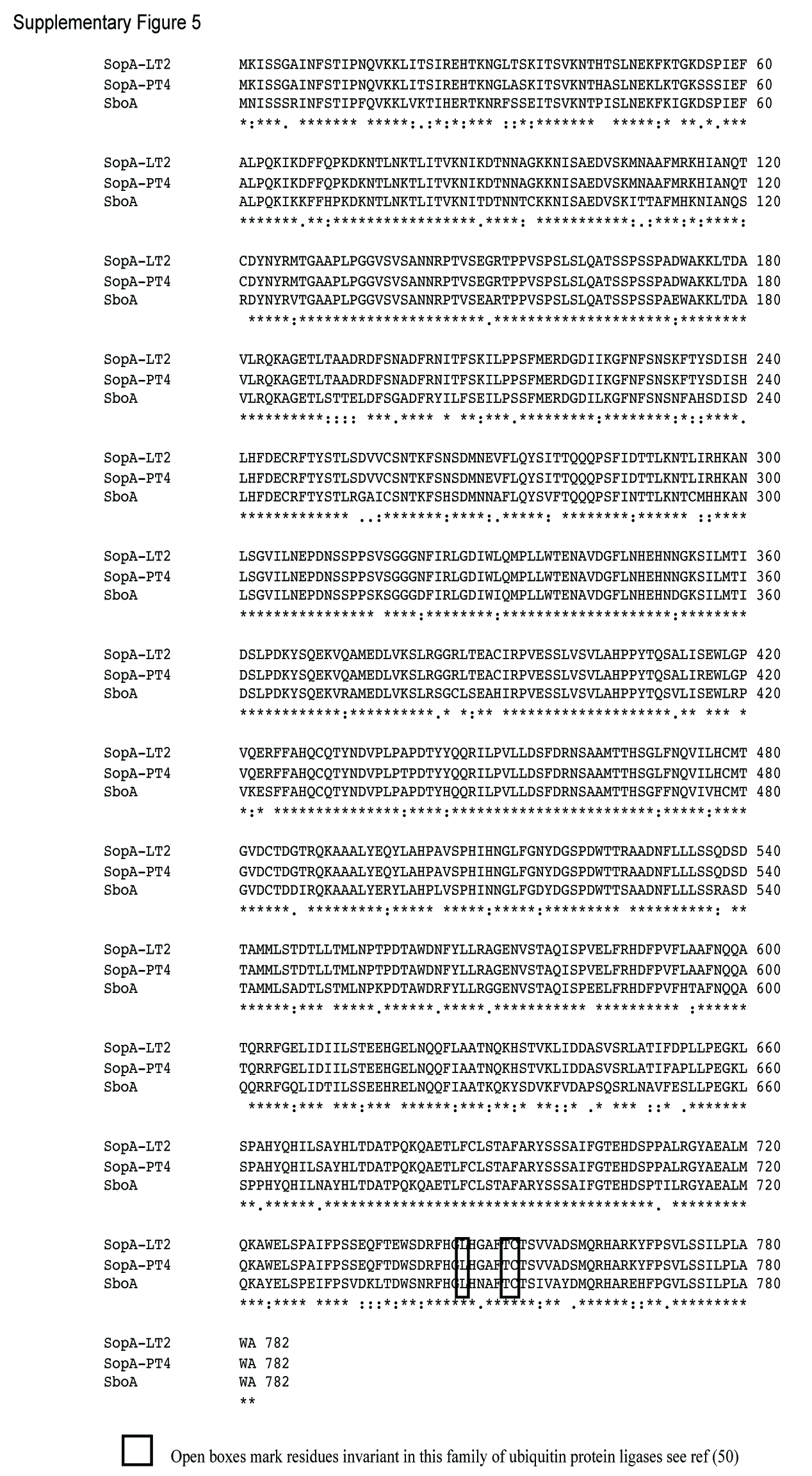

Supplement: Figure S5 — ClustalW alignment of the protein sequences of SopA from S. Typhimurium strains LT2 (SopA-LT2) and S. Enteritidis strain P125109 (SopA-PT4) with SboA from S. bongori strain 12419 (SboA). Conserved ubiquitin protein ligases domain is shown, see key. (TIF) [file ppat.1002191.s009.tif]
